# Supplementary material for: Enhanced coordination interaction with multi-site binding ligands for efficient and stable perovskite solar cells
Source: Nat Commun. 2025 Jul 11;16:6438. doi: 10.1038/s41467-025-61563-x (PMC12254260; doi:10.1038/s41467-025-61563-x)
Supplement: Supplementary file 1 — Supplementary Information [file 41467_2025_61563_MOESM1_ESM.pdf]

Supporting Information

Enhanced Coordination Interaction with Multi-Site Binding Ligands for Efficient and Stable Perovskite Solar Cells

Riming Nie,[*#1] Peikun Zhang,*[#1] *Jiaxing Gao,*[#1] *Cheng Wang,*[#1] *Weicun Chu,*[1] *Luyao Li,*[2] *Kaiyu Wang,*[3] *Dongmin Qian,*[3] *Fanrong Lin,*[1] *Xuefeng Xia,*[4] *Yong Wu,*[5] *Lingfeng Chao,*[3] *Chunyang Miao,*[3] *Xiaoming Zhao,*[1] *Wanlin Guo,*[*1] *and Zhuhua Zhang*[*1]

[1]State Key Laboratory of Mechanics and Control for Aerospace Structures, Key Laboratory for Intelligent Nano Materials and Devices of the Ministry of Education, and Institute for Frontier Science, Nanjing University of Aeronautics and Astronautics, Nanjing 210016, P. R. China

[2]School of Materials Science and Engineering, Shaanxi University of Science & Technology, Xi'an, 710021, P. R. China

[3]State Key Laboratory of Flexible Electronics (LoFE) & Institute of Advanced Materials (IAM), School of Flexible Electronics (Future Technologies), Nanjing Tech University (NanjingTech), Nanjing, 211816, China

[4]School of Electrical Engineering, Nanchang Institute of Technology, 289 Tianxiang Avenue, Nanchang, Jiangxi, 330099, China

[5]College of Mechanical and Electrical Engineering, Nanjing University of Aeronautics and Astronautics, Nanjing 210016, P. R. China

# These authors contribute equally to this work: Riming Nie, Peikun Zhang, Jiaxing Gao, Cheng Wang

E-mail: rmnie@nuaa.edu.cn; chuwazhang@nuaa.edu.cn; wlguo@nuaa.edu.cn

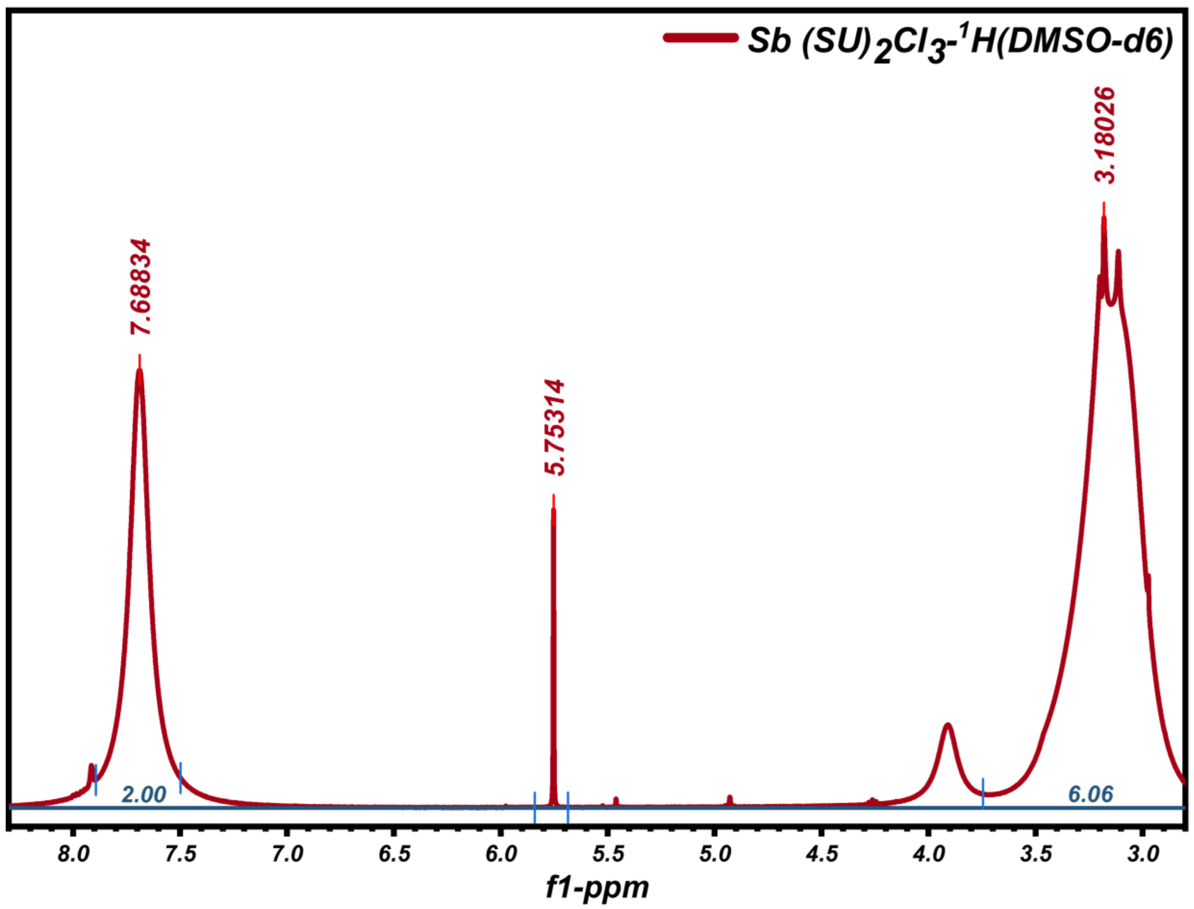

Supplementary Figure 1. Structural characterization. ^{1}H NMR spectrum of $Sb(SU)_2Cl_3$.

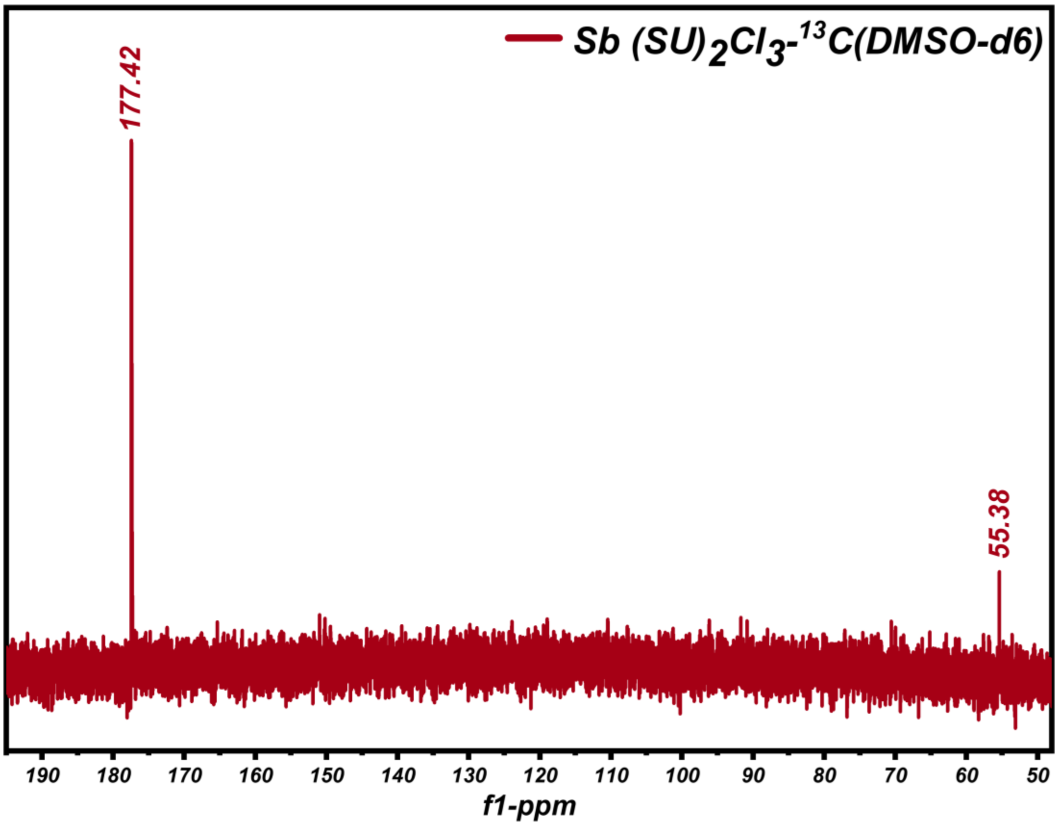

Supplementary Figure 2. Structural characterization. ^{13}C NMR spectrum of $Sb(SU)_2Cl_3$.

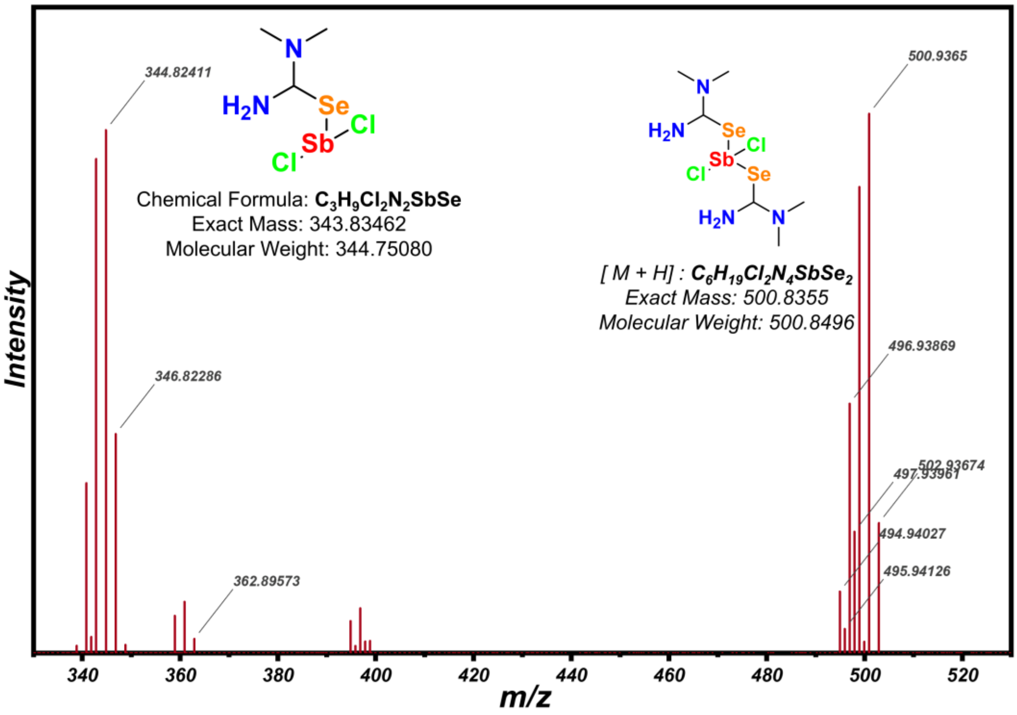

Supplementary Figure 3. Structural characterization. Mass spectrum of $Sb(SU)_2Cl_3$.

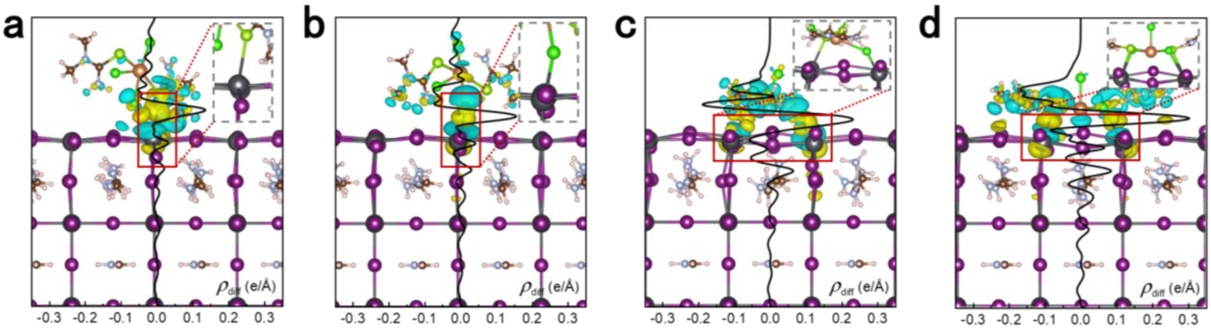

Supplementary Figure 4. Charge density difference. Charge density difference between $Sb(SU)_2Cl_3$ and the surface of PbI_2 termination, and corresponding charge displacement profiles for (a) Se adsorption site, (b) Cl adsorption site, (c) Se-Cl adsorption sites, and (d) double Se-Cl adsorption sites. The insets show the Pb-X bond (X = Se or Cl). The charge accumulation and depletion regions are distinguished by yellow and blue, respectively.

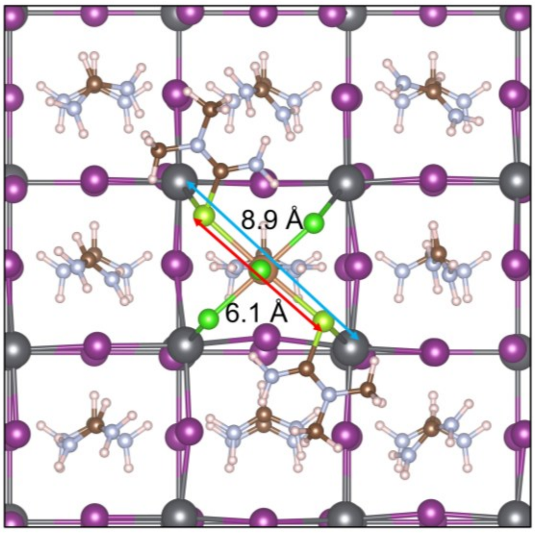

Supplementary Figure 5. Match information. The top view of the $Sb(SU)_2Cl_3$ treated $FAPbI_3$ surface.

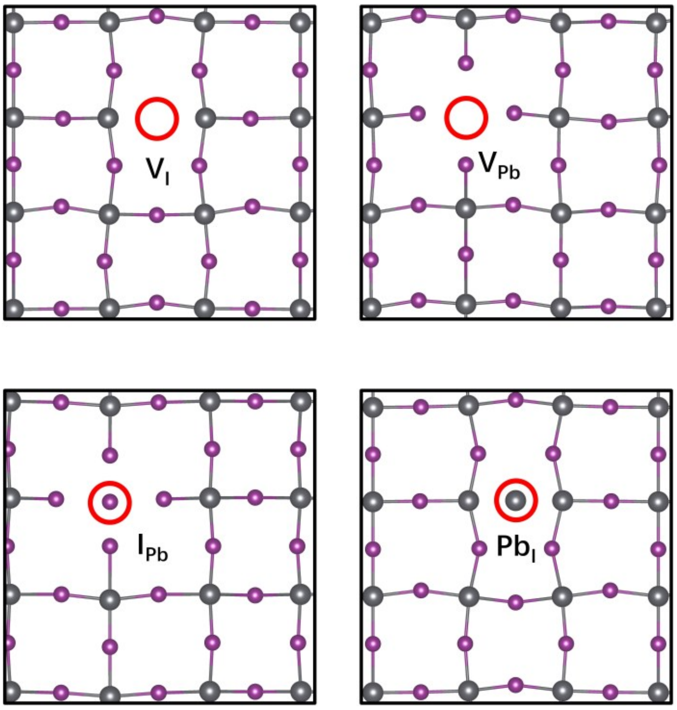

Supplementary Figure 6. Defects schematic diagram. Top view of the various types of surface point defects.

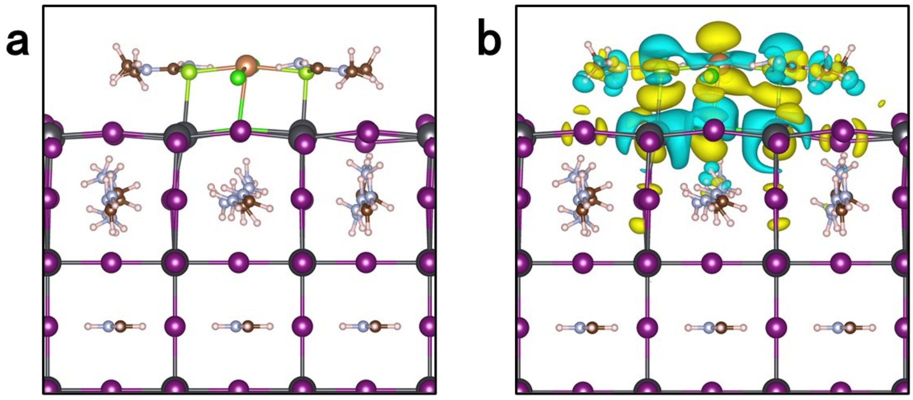

Supplementary Figure 7. The passivation of defects. Structure (a) and charge density difference (b) of Cl atom in the molecular filling I vacancy. The charge accumulation and depletion regions are distinguished by yellow and blue, respectively.

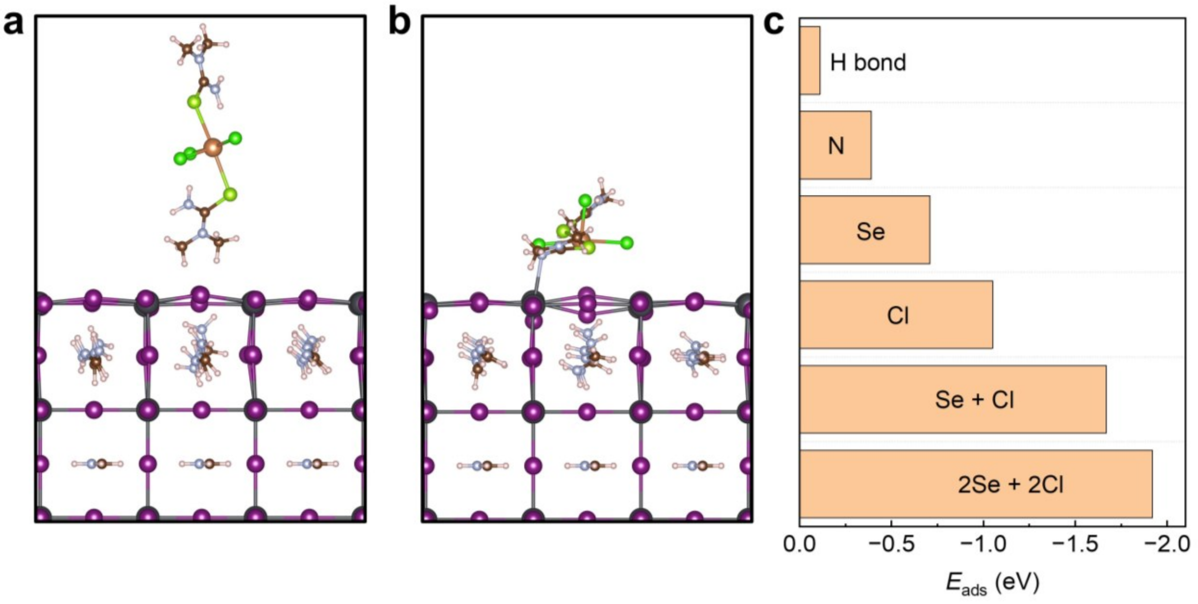

Supplementary Figure 8. The H atom's and N atom's passivation to the perovskite. Side views of the hydrogen bond interactions (a) and the N-Pb interactions (b) between the molecule and the perovskite surface. (c) The adsorption energies of six different interactions between the molecule and the perovskite surface.

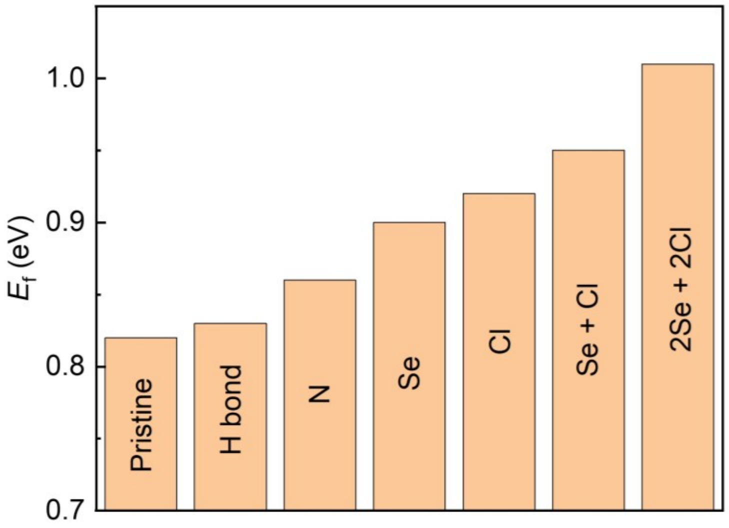

Supplementary Figure 9. Defect formation energies. Defect formation energies of V_I for the pristine perovskite and after the $Sb(SU)_2Cl_3$ molecule modified at six different sites.

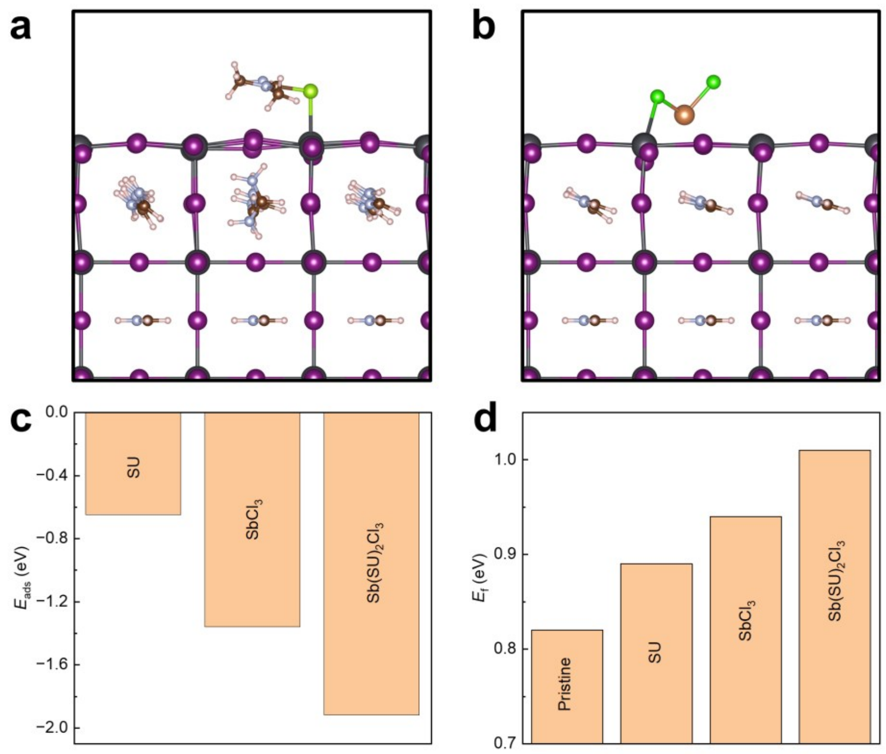

Supplementary Figure 10. The passivation of SU and $SbCl_3$ to the perovskite. Side views of the adsorption structures of the SU molecule (a) and the $SbCl_3$ molecule (b) on the perovskite surface. (c) The adsorption energies of the SU molecule, the $SbCl_3$ molecule, and the $Sb(SU)_2Cl_3$ molecule on the perovskite surface. (d) Defect formation energies of V_I for the perovskite surface after being modified by the SU molecule, the $SbCl_3$ molecule, and the $Sb(SU)_2Cl_3$ molecule.

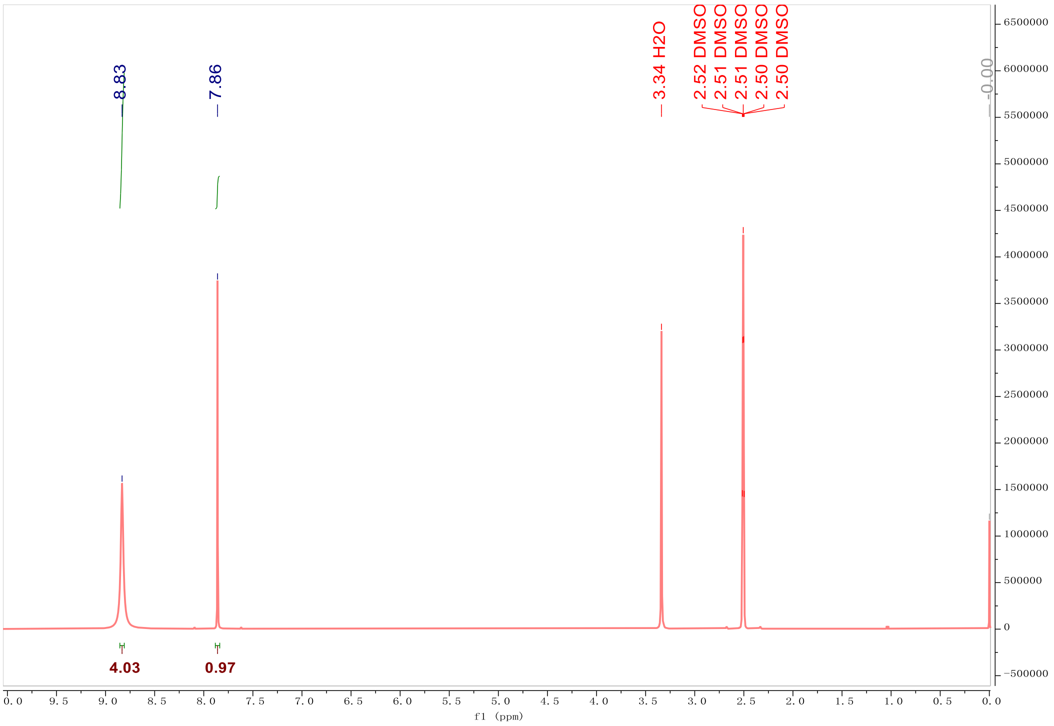

Supplementary Figure 11. Precursor interaction. Raw data for ^{1}H NMR spectra of FAI without $Sb(SU)_2Cl_3$.

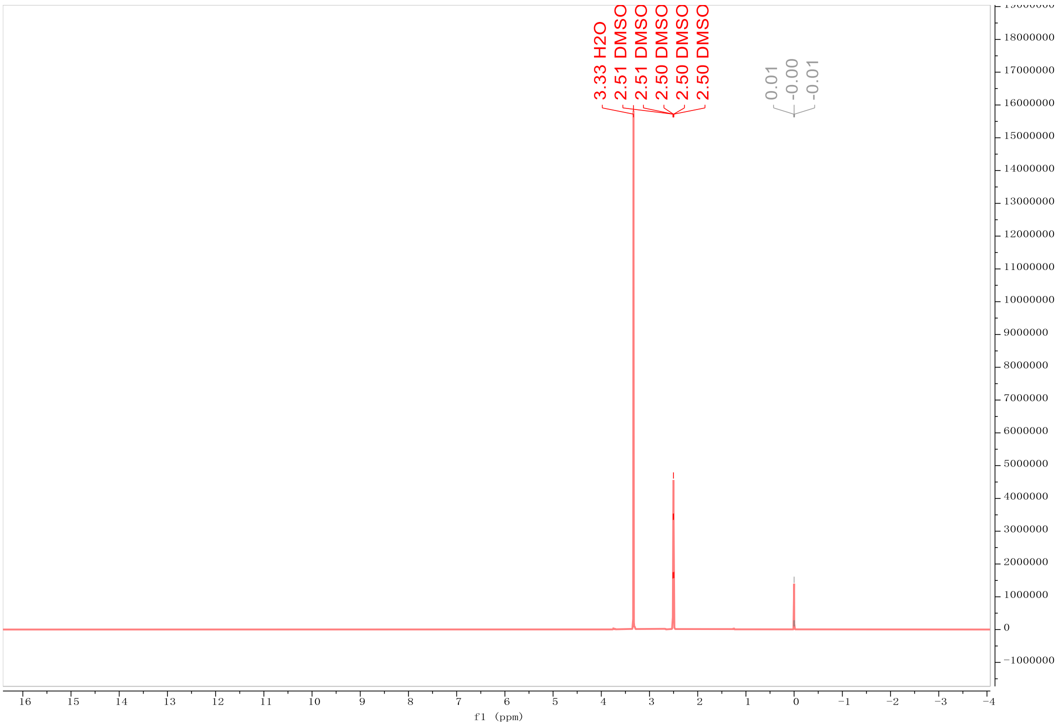

Supplementary Figure 12. Precursor interaction. Raw data for ^{1}H NMR spectra of PbI_2 without $Sb(SU)_2Cl_3$.

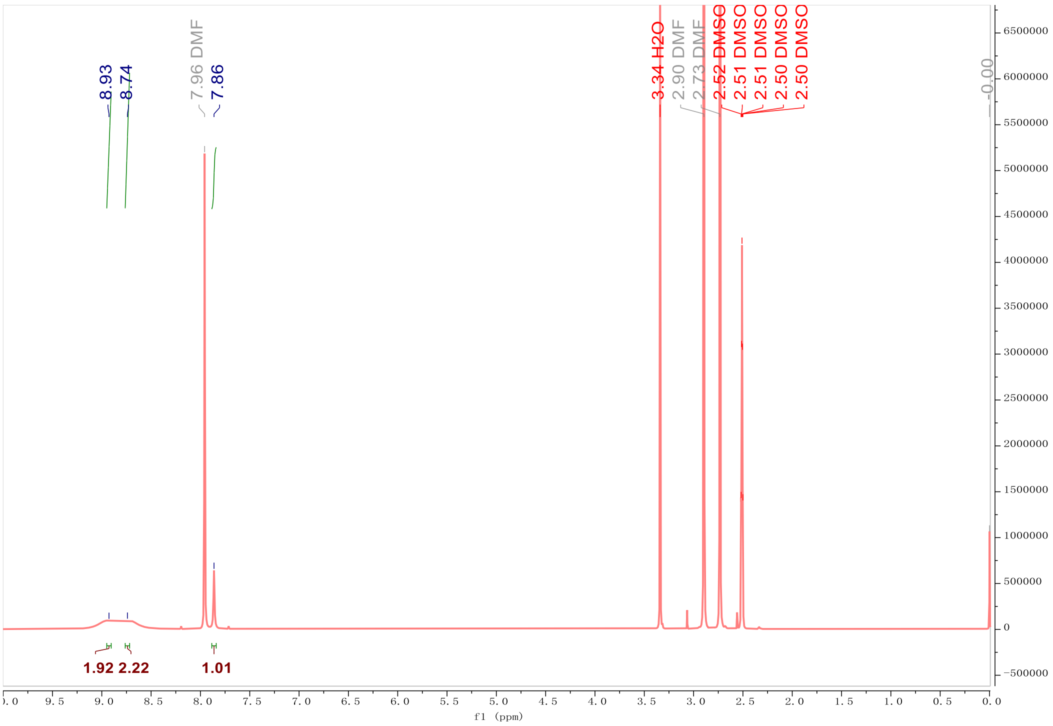

Supplementary Figure 13. Precursor interaction. Raw data for ^{1}H NMR spectra of $FAPbI_3$ without $Sb(SU)_2Cl_3$.

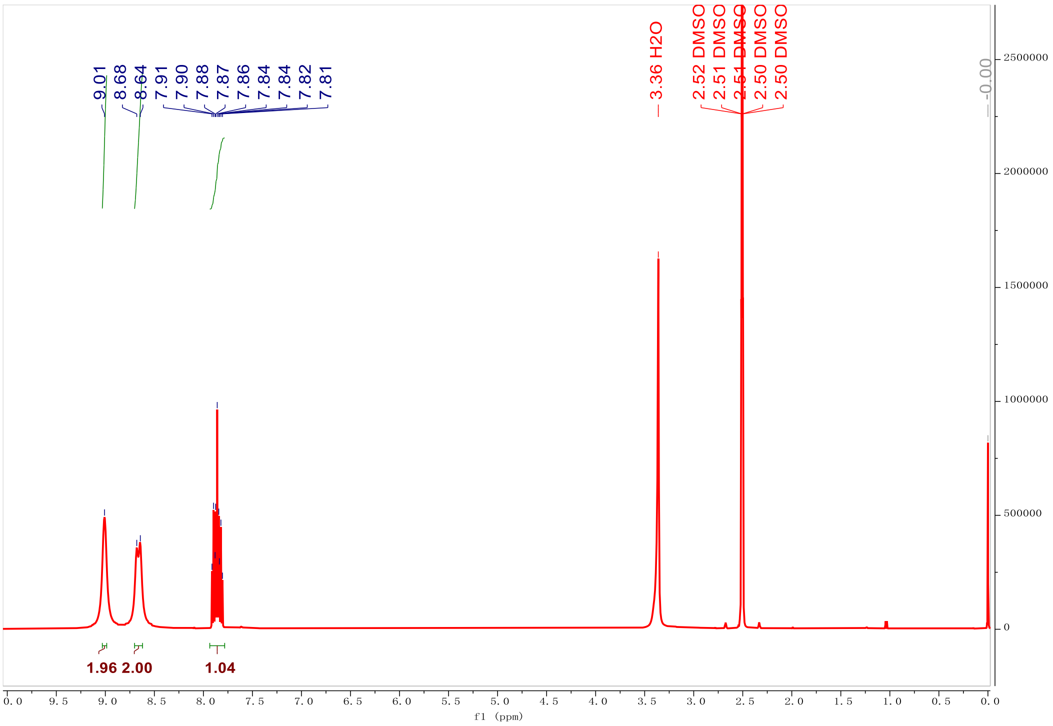

Supplementary Figure 14. Precursor interaction. Raw data for ^{1}H NMR spectra of FAI with $Sb(SU)_2Cl_3$.

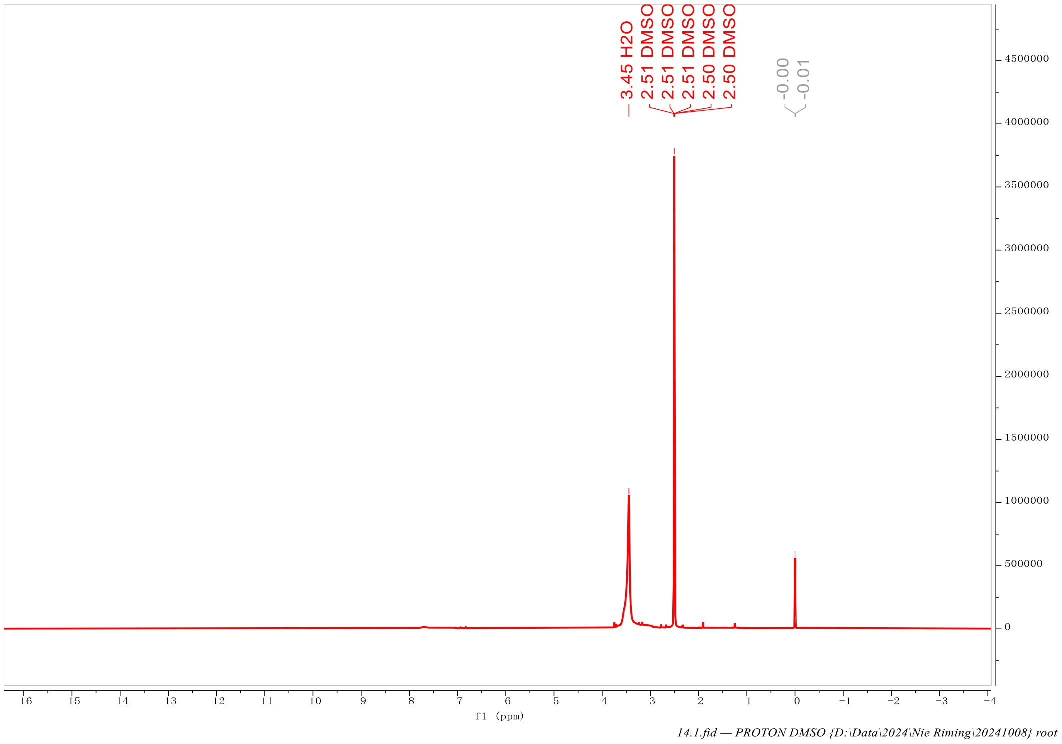

Supplementary Figure 15. Precursor interaction. Raw data for ^{1}H NMR spectra of PbI_2 with $Sb(SU)_2Cl_3$.

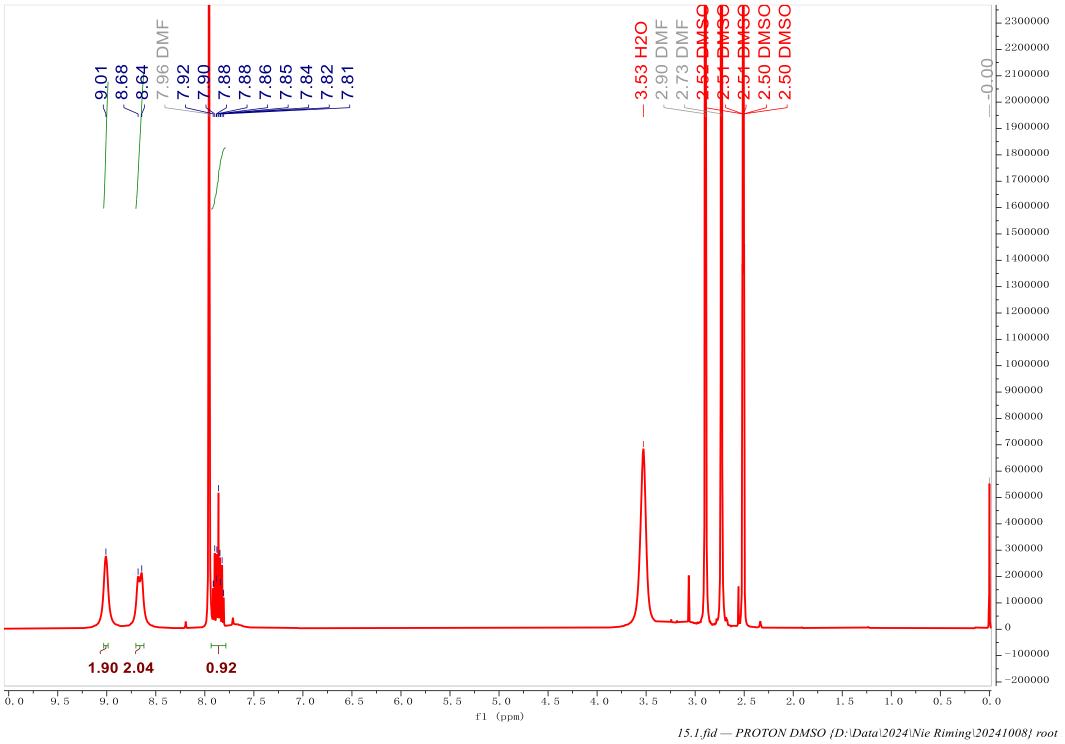

Supplementary Figure 16. Precursor interaction. Raw data for ^{1}H NMR spectra of $FAPbI_3$ with $Sb(SU)_2Cl_3$.

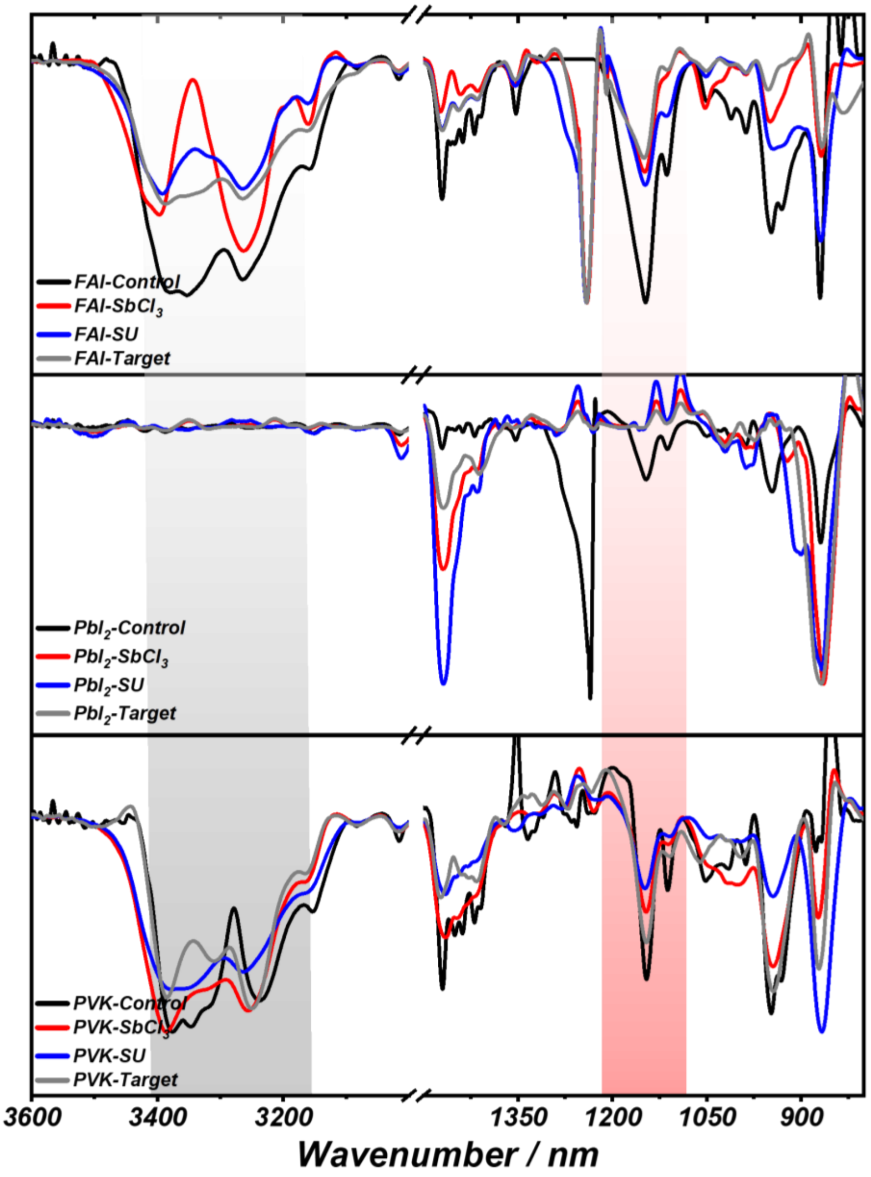

Supplementary Figure 17. Fourier infrared absorption spectra. Fourier infrared absorption spectroscopy was performed on FAI, PbI_2 and PVK for the control unit and monomer, respectively.

To probe molecular-level interactions, we performed Fourier-transform infrared (FTIR) spectroscopy on individual components (SU, $SbCl_3$, and $Sb(SU)_2Cl_3$) and their mixtures with FAI, PbI_2, and perovskite (PVK) films. In the FAI system, the N-H stretching vibration (3200-3400 cm^{-1}) showed progressive red shifts: from 3280 cm^{-1} (FAI-Control) to 3265 cm^{-1} (FAI-SU), 3250 cm^{-1} (FAI-Sb), and 3235 cm^{-1} (FAI-Target), suggesting that $Sb(SU)_2Cl_3$ forms the most stable hydrogen-bond network with FA^+, thus enhancing structural stability. In the PbI_2 system, the Pb-I stretching band (1350-1250 cm^{-1}) red-shifted from 1290 cm^{-1} (PbI_2-Control) to 1282 cm^{-1} (PbI_2-SU), 1270 cm^{-1} (PbI_2-Sb), and 1255 cm^{-1} (PbI_2-Target), indicating that the complex exhibits the strongest coordination with Pb^{2+}, possibly due to the enhanced binding

induced by Cl ligands. For the perovskite films, both N-H and Pb-I vibrations showed significant shifts: from 3275 cm^{-1} to 3225 cm^{-1} (N-H) and from 1180 cm^{-1} to 1135 cm^{-1} (Pb-I), confirming that Cl coordinates with Pb^{2+} to stabilize the perovskite lattice. Mechanistic analysis suggests SU interacts with FA^+ via hydrogen bonding, while $SbCl_3$ acts as a Lewis acid to enhance electrostatic interactions. The $Sb(SU)_2Cl_3$ exhibits the strongest binding in all cases due to its multidentate interaction capability. These findings highlight the critical role of Cl in stabilizing both precursor and perovskite structures, providing a rational basis for improving film quality and reducing defect states.

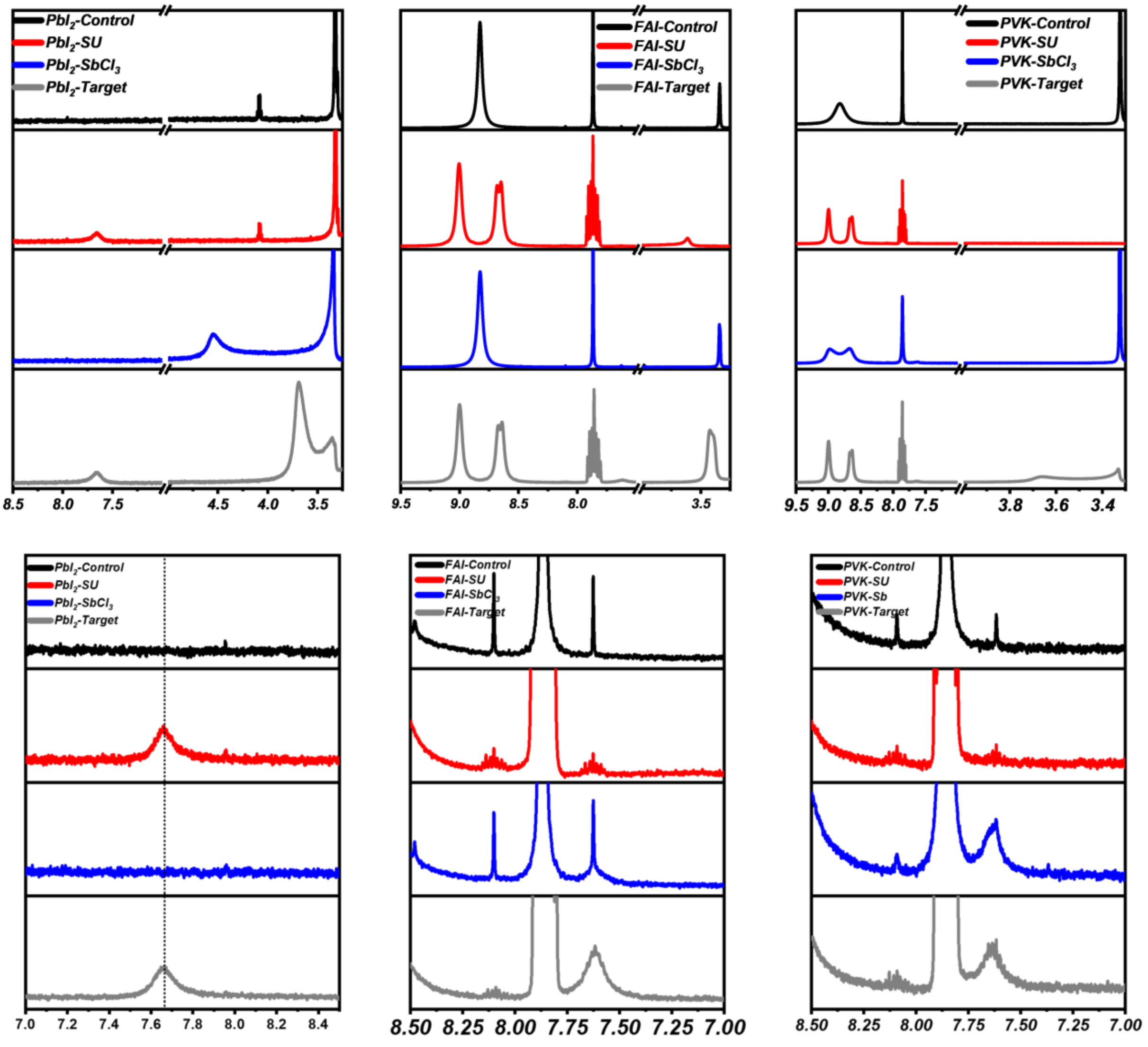

Supplementary Figure 18. Liquid NMR spectra. Liquid NMR spectroscopy was performed on the control units and monomers of FAI, PbI_2 and PVK, respectively.

^1H nuclear magnetic resonance (NMR) spectroscopy was performed to probe interactions between the three additives and FAI, PbI_2, and PVK in solution. In the PbI_2 system, no distinct

hydrogen signals were observed in the 7-9 ppm region for PbI_2 and PbI_2-$SbCl_3$, suggesting $SbCl_3$ primarily coordinates with Pb^{2+} via Cl without perturbing the proton environment. In contrast, PbI_2-SU and PbI_2-Target displayed significant signal enhancements, indicating interactions through selenourea (SU) or N-H groups. The stronger signals in the PbI_2-Target system suggest more stable coordination networks involving both Cl and SU functionalities. In the FAI system, the intrinsic N-H signals (8.9-9.5 ppm) exhibited chemical shifts depending on the additive. No substantial shift was seen for FAI-$SbCl_3$, whereas notable downfield shifts occurred in FAI-SU and especially FAI-Target, indicating enhanced hydrogen bonding and possible FA^+-PbI_2 interactions. Furthermore, the FAI-Target exhibited stronger C-H related signals in the 7-8 ppm region than FAI-SU and FAI-$SbCl_3$, suggesting multidentate coordination that stabilizes both PbI_2 and FA^+ species. In the PVK system, downfield shifts of the FA^+ N-H signal were observed in the PVK-$SbCl_3$ and PVK-Target systems. Notably, the PVK-Target sample displayed stronger N-H peaks and more pronounced low-field shifts, indicating persistent multidentate interactions in the solid-state film. These interactions may involve: (1) direct Cl coordination with Pb^{2+} to enhance structural stability; (2) SU-mediated hydrogen bonding with PbI_2 or FA^+ to reinforce precursor-cation interactions; and (3) N-H…X (X = I, Se) hydrogen bonding to anchor FA^+ and restrict its mobility, potentially enhancing long-term stability.

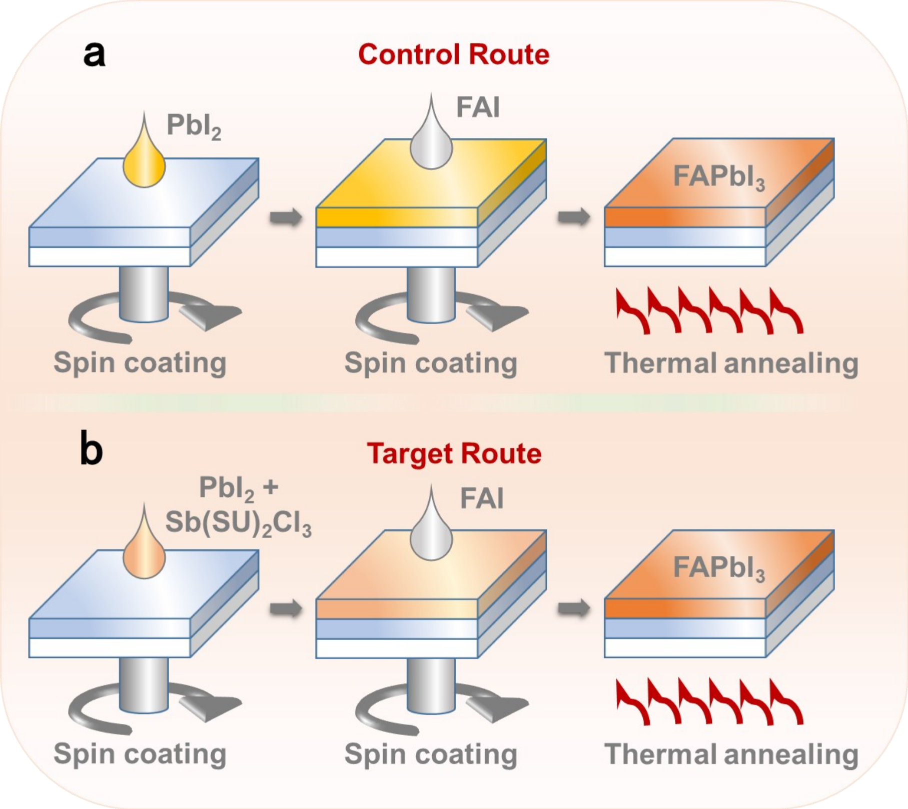

Supplementary Figure 19. Control and Target routes. a) Control route (without $Sb(SU)_2Cl_3$) and b) Target route (with $Sb(SU)_2Cl_3$) for preparing the perovskite film.

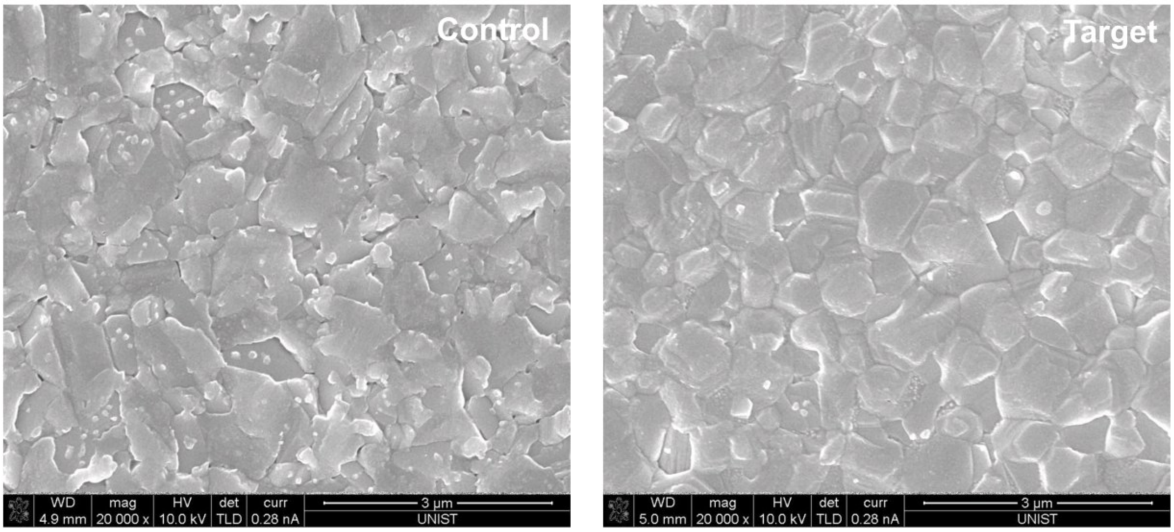

Supplementary Figure 20. Morphology. SEM images of the control and target perovskite film.

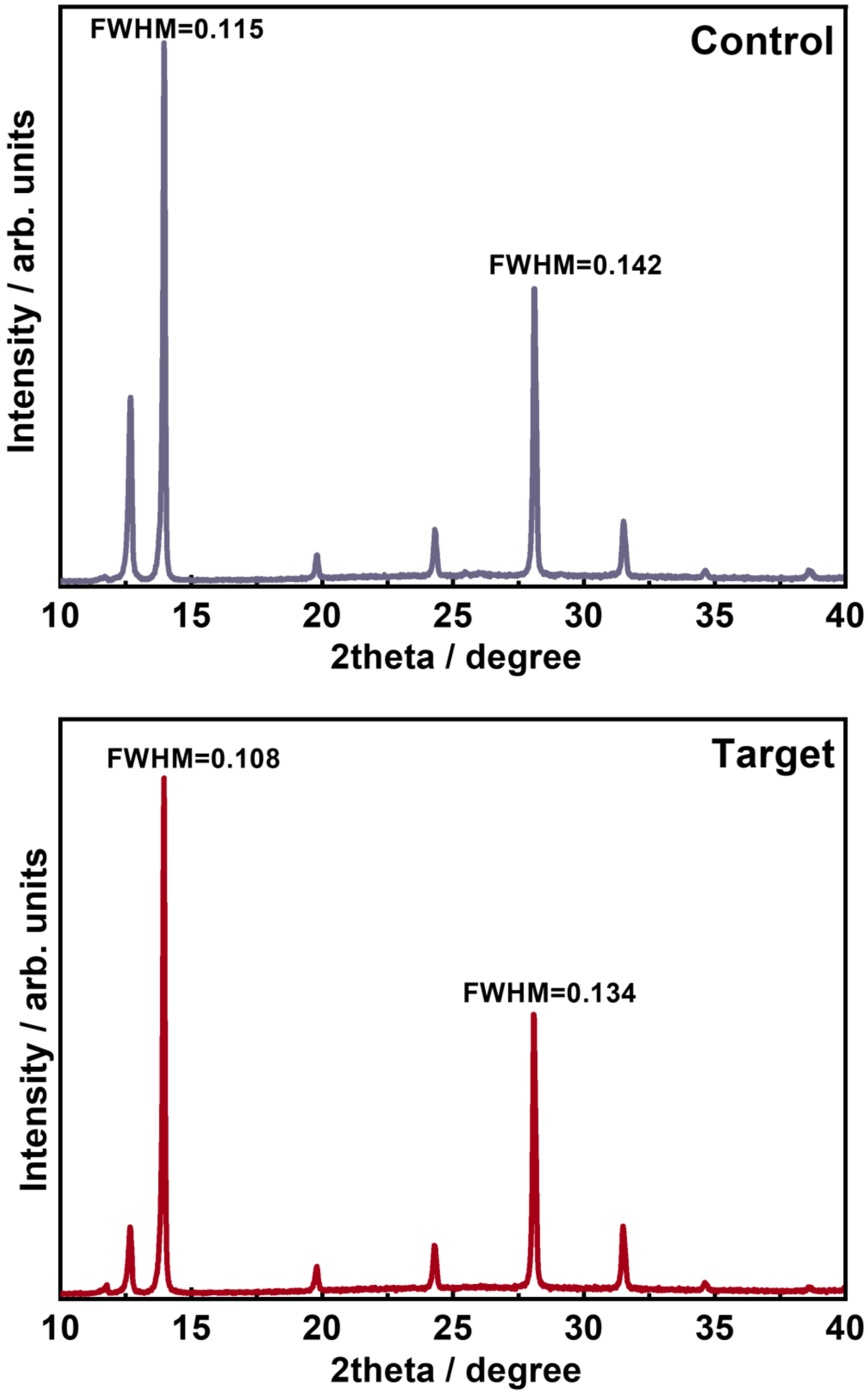

Supplementary Figure 21. Crystallization. XRD of the control and target perovskite films.

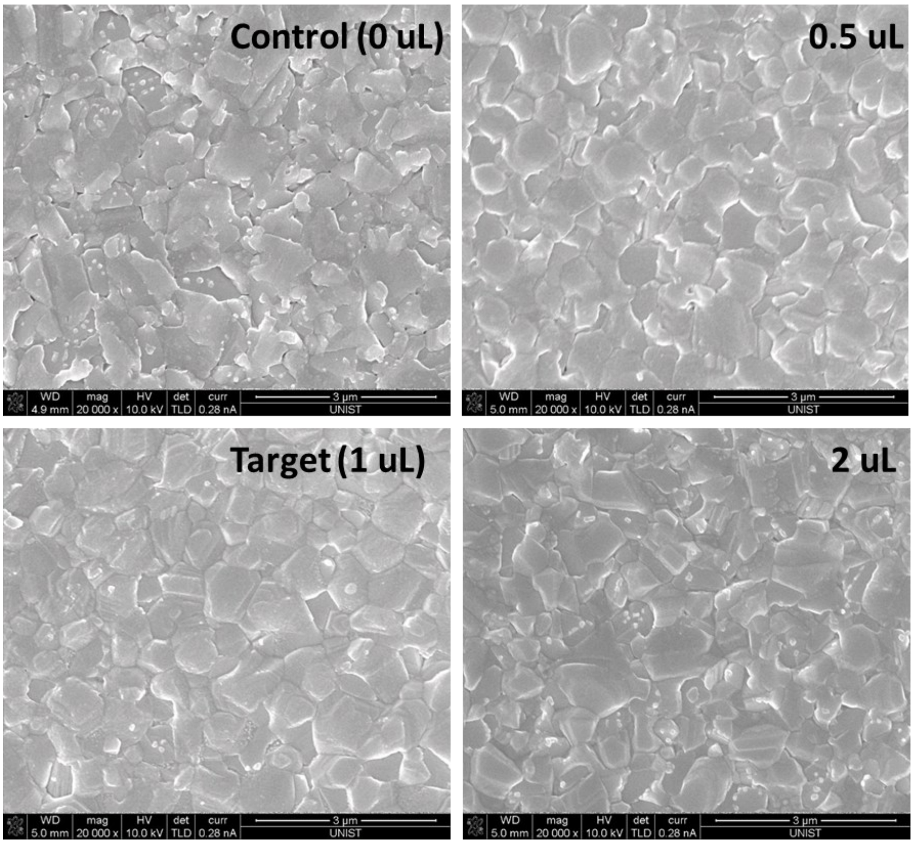

Supplementary Figure 22. Morphology. Surface SEM images of the perovskite films with various amount of $Sb(SU)_2Cl_3$.

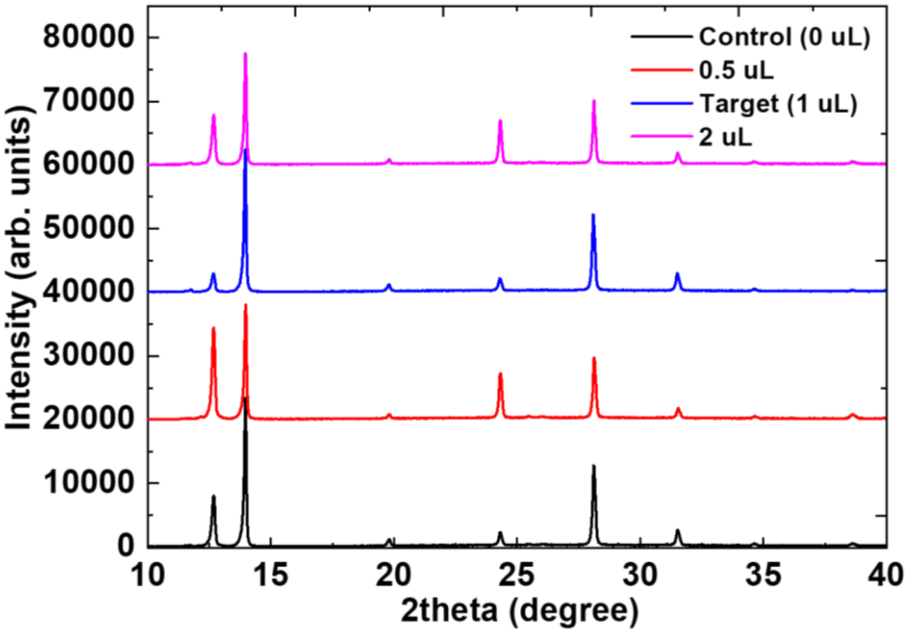

Supplementary Figure 23. Crystallization. XRD patterns of the perovskite films with various amount of $Sb(SU)_2Cl_3$.

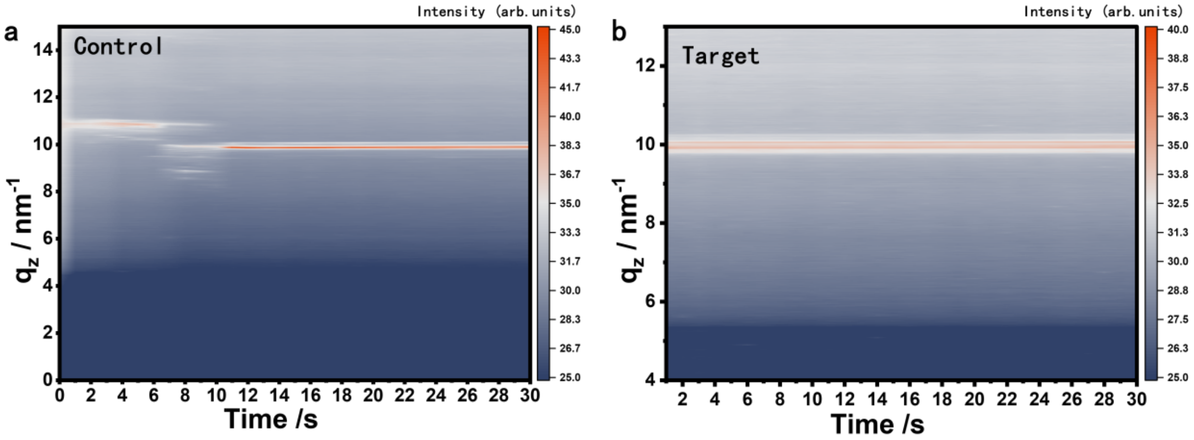

Supplementary Figure 24. Crystallization kinetics. In situ GIWAX in the control group (a) and target group (b)

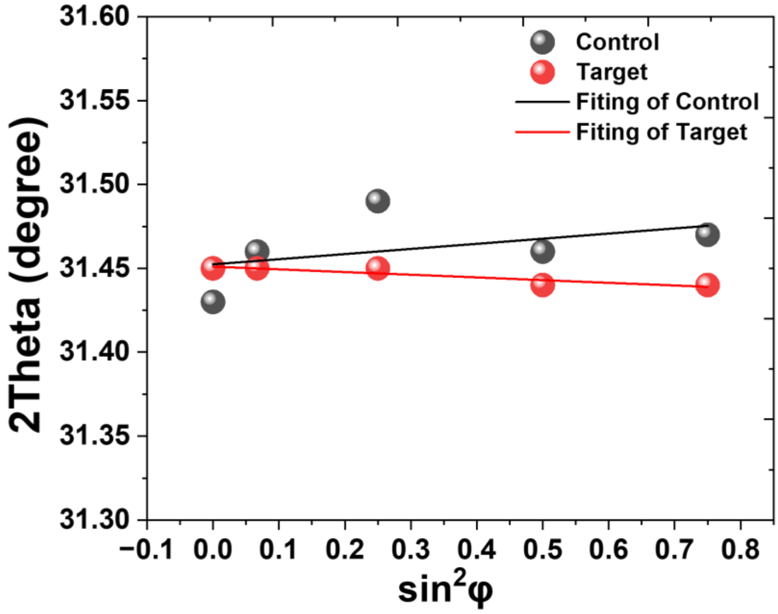

Supplementary Figure 25. Strain. Linear fit of 2θ-sin^{2}ϕ.

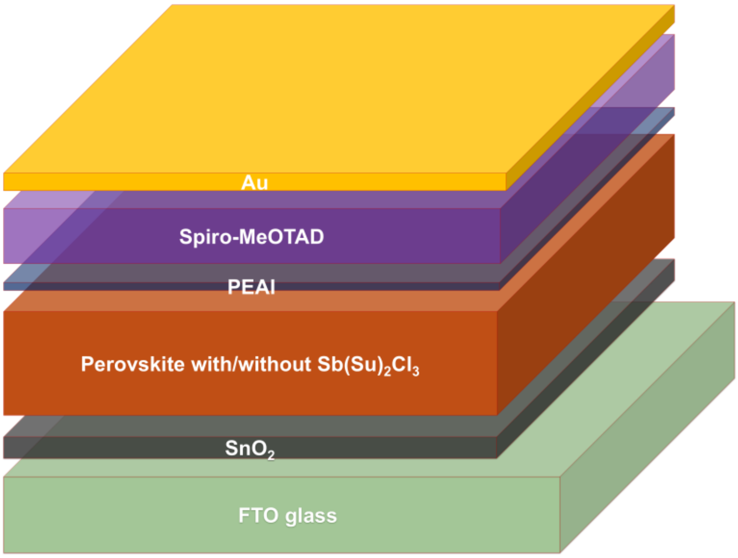

Supplementary Figure 26. Device structure. Device structure of the perovskite solar cells with and without $Sb(SU)_2Cl_3$.

Report No. 24TR012002

====Measurement Results ====

	Forward Scan (Isc to Voc)	Reverse Scan (Voc to Isc)
Area	4.40 mm^2	
Isc	1.079 mA	1.079 mA
Voc	1.183 V	1.184 V
Pmax	1.069 mW	1.071 mW
Ipm	1.043 mA	1.046 mA
Vpm	1.025 V	1.024 V
FF	83.72 %	83.84 %
Eff	24.29 %	24.34 %

- Active area was provided by client.
- Test results listed in this measurement report refer exclusively to the mentioned measured sample.
- The results apply only at the time of the test, and do not imply future performance.

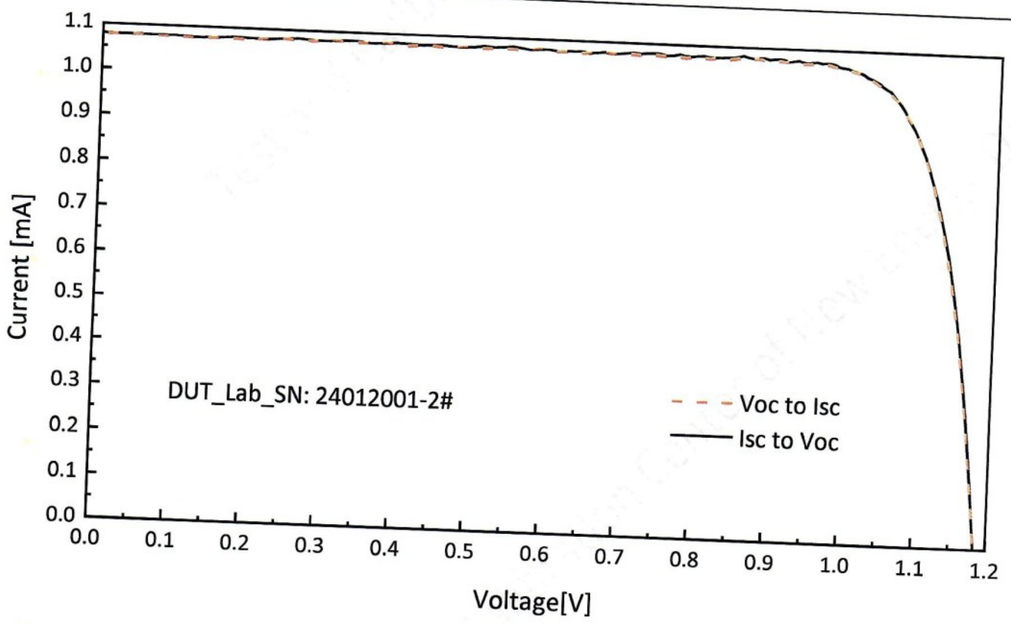

Fig.1 I-V curves of the measured sample

-------**End of Report**-------

Supplementary Figure 27. Certificated cell. Certificated results from an accredited photovoltaic certification laboratory (Shanghai Institute of Microsystem and Information Technology Chinese Academy of Sciences (SlMlT)).

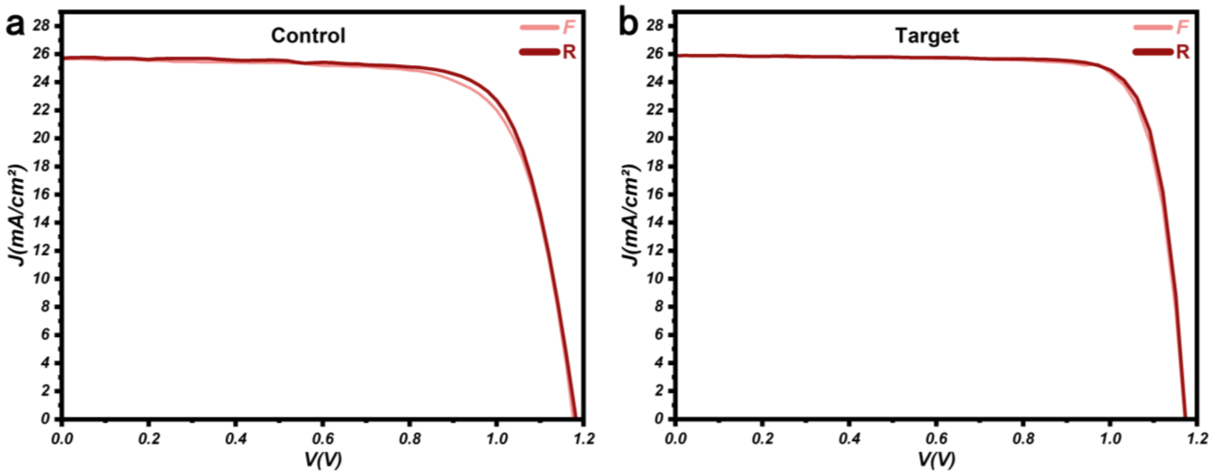

Supplementary Figure 28. Hysteresis. Current density-voltage curves of the a) control and b) target PSCs measured in the reverse and forward mode.

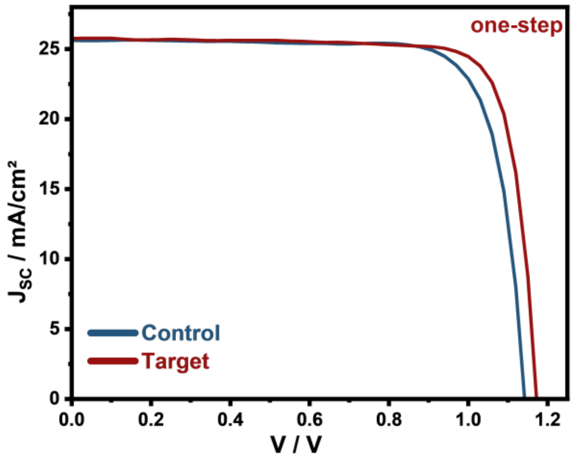

Supplementary Figure 29. One-step fabricated devices. J-V curves for one-step method with and without $Sb(SU)_2Cl_3$.

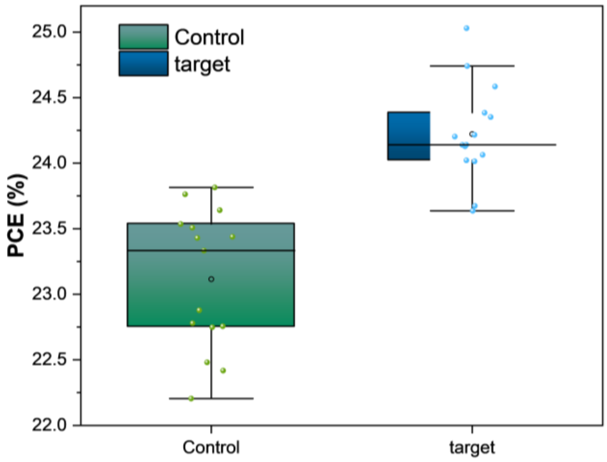

Supplementary Figure 30. Statistical analysis of PCEs. Statistical analysis across 15 independent control and target devices. The median value, 25 region of data, and 75 region of data are represented by the horizontal line (across the box), bottom line, and top line of the box. The mean, maximum and minimum values are represented by the circle, top and bottom bars, respectively.

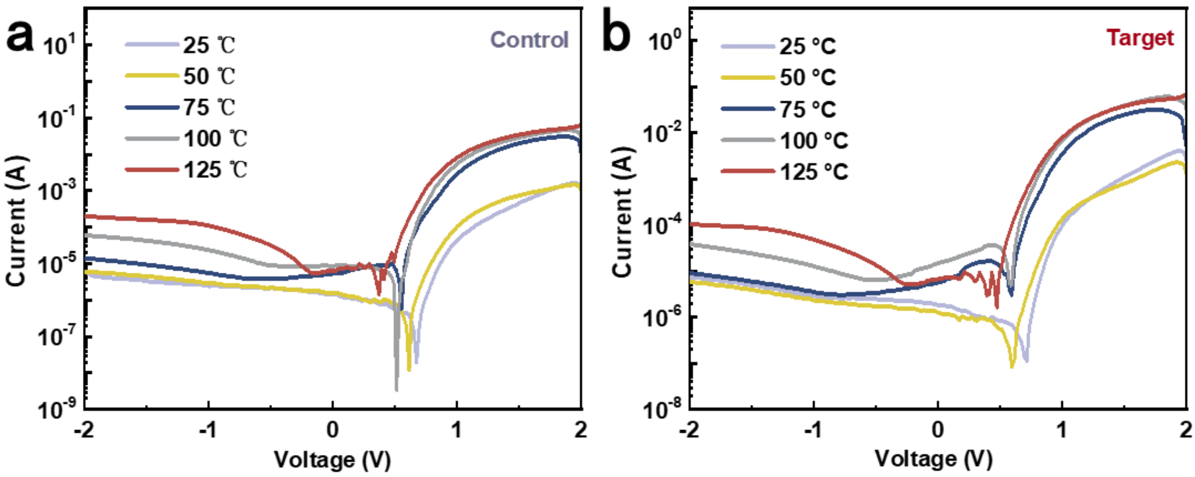

Supplementary Figure 31. Temperature dependent dark current-voltage curves. a) and b) Current-voltage curves in the dark of the control and target perovskite solar cells.

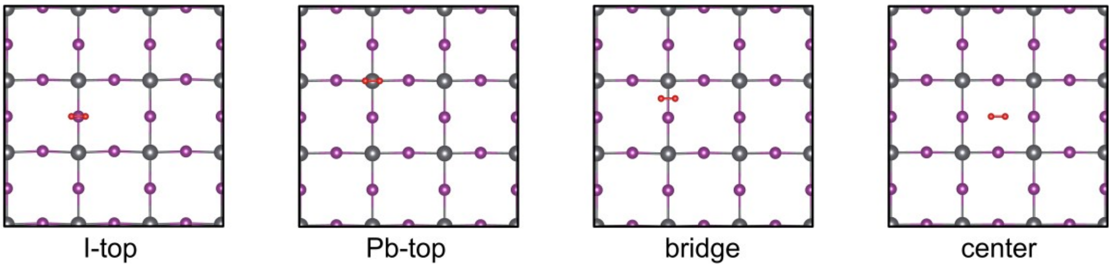

Supplementary Figure 32. Adsorption sites for O_2. O_2 adsorption at different sites on $FAPbI_3$ (100) surface.

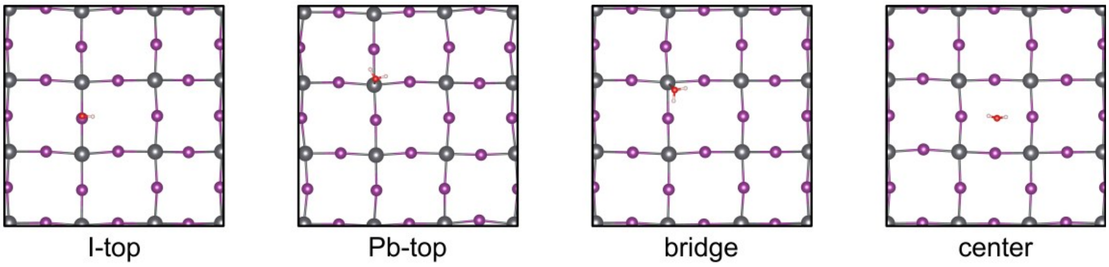

Supplementary Figure 33. Adsorption sites for H_2O. H_2O adsorption at different sites on $FAPbI_3$ (100) surface.

Supplementary Table 1. The data extracted from Supplementary Figure 4d. Here R_s denotes series resistance, R_{trans} and C_{trans} denote the resistance and capacitance associated with the hole transport material (HTM), and R_{rec} and C_{rec} denote the resistance and capacitance at the perovskite/HTM interface.

	R_s (Ω)	R_{trans} (Ω)	C_{trans}	R_{rec} (Ω)	C_{rec}
**Ref**	92	261.3	4.5083e-8	1640	6.7117e-8
**0.5**	76	63	3.6598e-5	2800	2.2861e-8
**1**	48	57	3.0089e-7	3950	1.8523e-8
**2**	112	105	1.1548e-7	1750	3.1469e-8

Supplementary Table 2. Summary of high-efficiency (PCE > 22%) thermal stability.

Perovskite	Processing Method	temperature	Relative Humidity (%)	stability	References
**$FAPbI_3$**	Two-step	85	-	$T_{97.2}$=1400h	This Work
**$FAPbI_3$**	Two-step	85	-	T_{99}=1100h	Ref. 1
**$FAPbI_3$**	Two-step	85	85	T_{70}=1000h	Ref. 2
**$FAPbI_3$**	One-step	85	-	T_{80}=700h	Ref. 3

Supplementary Table 3. Summary of high-efficiency (PCE > 22%) MPPT stability**.**

Perovskite	Processing Method	temperature	stability	References
**$FAPbI_3$**	**Two-step**	25	**$T_{92.2}$=2000h**	**This Work**
$FAPbI_3$	One-step	-20	T_{90}=1000h	Ref. 4
$FA_{0.95}Cs_{0.05}PbI_3$	Two-step	30	T_{95}=1000h	Ref. 5
$FAPbI_3$	Two-step	50	T_{94}=1258h	Ref. 6

Supplementary Table 4. A summary of the efficiency of perovskite solar cells prepared by all air method reported in relevant literature and this work.

**Perovskite**	Champion PCE	Relative humidity	References
**$FAPbI_3$**	**25.03**	**20-40**	**This Work**
**$FAPbI_3$**	24.70	20	Ref. 7
**$FAPbI_3$**	25.70	20	Ref. 8
**$FAMAPbI_3$**	24.45	35-45	Ref. 9

Supplementary Table 5. Adsorption energies of O_2 on the $FAPbI_3$ (100) surfaces with and without treatment.

O_2	I-top	Pb-top	bridge	center
$FAPbI_3$	-0.196	-0.262	-0.244	-0.137
Treated-$FAPbI_3$	-0.213	-0.247	-0.24	-0.025

Supplementary Table 6. Adsorption energies of H_2O on the $FAPbI_3$ (100) surfaces with and without treatment.

H_2O	I-top	Pb-top	bridge	center
$FAPbI_3$	-0.338	-0.598	-0.596	-0.418
Treated-$FAPbI_3$	-0.292	-0.454	-0.467	-0.436

Supplementary References :

[1] Yu Zhang, Yanrun Chen, Guilin Liu, Nonalloyed α-phase formamidinium lead triiodide solar cells through iodine intercalation, Science, 2025, 387(6731): 284-290.

[2] Lingbo Xiao, Xiaoli Xu, Jie Zhao, High-Temperature Driven Recrystallization for Stable Dopant-Free α-$FAPbI_3$ Perovskite Solar Cells, Advanced Science, 2024, 11(48): 2408684.

[3] Lusheng Liang, Zi-Ang Nan, Yuheng Li, Formation Dynamics of Thermally Stable 1D/3D Perovskite Interfaces for High-Performance Photovoltaics, Advanced Material, 2025, 37(8):2413841

[4] Muyang Chen, Tingting Niu, Lingfeng Chao, "Freezing" intermediate phases for efficient and stable $FAPbI_3$ perovskite solar cells, Energy Environ. Sci., 2024,17, 3375-3383.

[5] Pengju Shi, Yong Ding, Bin Ding, Oriented nucleation in formamidinium perovskite for photovoltaics, Nature, 2023, 620: 323-327.

[6] Zijian Huang, Yang Bai, Xudan Huang, Anion-π interactions suppress phase impurities in $FAPbI_3$ solar cells, Nature, 2023, 623: 531-537.

[7] Zou Y, Yu WJ, Guo HQ, Li QZ, Li XD, Li L, et al. A crystal capping layer for formation of black-phase $FAPbI_3$ perovskite in humid air. Science. 2024; 385(6705) 161-167.

[8] Yang Y, Huang H, Yan L, Cui P, Lan Z, Sun C, et al. Compatible Soft-Templated Deposition and Surface Molecular Bridge Construction of SnO_2 Enable Air-Fabricated Perovskite Solar Cells with Efficiency Exceeding 25.7%. Advanced Energy Materials. 2024; 14(23) 2400416.

[9] Zeng Q, Xiao H, Ma Q, Huang R, Pan Y, Li L, et al. Highly Layer-Oriented PbI_2 Films Enabling All-Air Processed Perovskite Solar Cells. Advanced Energy Materials. 2024; 14(32) 2401279.
