# Supplementary material for: Enhanced coordination interaction with multi-site binding ligands for efficient and stable perovskite solar cells
Source: Nat Commun. 2025 Jul 11;16:6438. doi: 10.1038/s41467-025-61563-x (PMC12254260; doi:10.1038/s41467-025-61563-x)
Supplement: Supplementary file 2 — Reporting Summary [file 41467_2025_61563_MOESM2_ESM.pdf]

nature portfolio

Corresponding author(s): Wanlin Guo, Zhuhua Zhang, Riming Nie

Solar Cells Reporting Summary

Nature Portfolio wishes to improve the reproducibility of the work that we publish. This form is intended for publication with all accepted papers reporting the characterization of photovoltaic devices and provides structure for consistency and transparency in reporting. Some list items might not apply to an individual manuscript, but all fields must be completed for clarity.

For further information on Nature Research policies, including our data availability policy, see Authors & Referees.

• Experimental design

Please check the following details are reported in the manuscript, and provide a brief description or explanation where applicable.

1. Dimensions

Item	Yes/No	Response
Area of the tested solar cells	[x] Yes [ ] No	0.094 cm2
		Explain why this information is not reported/not relevant.
Method used to determine the device area	[x] Yes [ ] No	light mask
		Explain why this information is not reported/not relevant.

2. Current-voltage characterization

Item	Yes/No	Response
Current density-voltage (J-V) plots in both forward and backward direction	[x] Yes [ ] No	Figure S27
Voltage scan conditions	[x] Yes [ ] No	[Measurement conditions]<br>Sweep direction: Reverse<br>NPLC: 0.01<br>Voltage settling time (s): 10.00m
		Explain why this information is not reported/not relevant.
Test environment	[x] Yes [ ] No	in the atmosphere (25 degree 20-40% humidity)
		Explain why this information is not reported/not relevant.
Protocol for preconditioning of the device before its characterization	[ ] Yes [x] No	*Provide a description of the protocol.*
		we did not do it
Stability of the J-V characteristic	[x] Yes [ ] No	under 1 sun illumination measured at Vmmp
		Explain why this information is not reported/not relevant.

3. Hysteresis or any other unusual behaviour

Item	Yes/No	Response
Description of the unusual behaviour observed during the characterization	[x] Yes [ ] No	Figure S27
		Explain why this information is not reported/not relevant.
Related experimental data	[ ] Yes [ ] No	*Provide a description of the related experimental data.*
		Explain why this information is not reported/not relevant.

4. Efficiency

Item	Yes/No	Response
External quantum efficiency (EQE) or incident photons to current efficiency (IPCE)	[x] Yes [ ] No	EQE spectrum was measured using an internal quantum efficiency system (Oriel, IQE 200B)
		Explain why this information is not reported/not relevant.

A comparison between the integrated response under the standard reference spectrum and the response measure under the simulator

- [x] Yes
- [ ] No

Page 14

Explain why this information is not reported/not relevant.

For tandem solar cells, the bias illumination and bias voltage used for each subcell

- [ ] Yes
- [ ] No

Provide a description of the measurement conditions.

Explain why this information is not reported/not relevant.

5. Calibration

Light source and reference cell or sensor used for the characterization

- [x] Yes
- [ ] No

A standard silicon cell is used for calibration

Explain why this information is not reported/not relevant.

Confirmation that the reference cell was calibrated and certified

- [x] Yes
- [ ] No

Calibration of China Metrology Institute

Explain why this information is not reported/not relevant.

Calculation of spectral mismatch between the reference cell and the devices under test

- [ ] Yes
- [ ] No

Provide a value of the spectral mismatch and/or a description of how it has been taken into account in the measurements.

Explain why this information is not reported/not relevant.

6. Mask/aperture

Size of the mask/aperture used during testing

- [x] Yes
- [ ] No

0.094 cm2

Explain why this information is not reported/not relevant.

Variation of the measured short-circuit current density with the mask/aperture area

- [x] Yes
- [ ] No

Report the difference in the short-circuit current density values measured with the mask and aperture area.

Explain why this information is not reported/not relevant.

7. Performance certification

Identity of the independent certification laboratory that confirmed the photovoltaic performance

- [ ] Yes
- [x] No

Identify the independent certification laboratory.

consider the time and cost of certification

A copy of any certificate(s)

- [ ] Yes
- [ ] No

Certificate copies should be provided in the Supplementary information. Please state the supplementary item number.

Explain why this information is not reported/not relevant.

8. Statistics

Number of solar cells tested

- [x] Yes
- [ ] No

50

Explain why this information is not reported/not relevant.

Statistical analysis of the device performance

- [ ] Yes
- [x] No

State where this information can be found in the text.

Explain why this information is not reported/not relevant.

9. Long-term stability analysis

Type of analysis, bias conditions and environmental conditions

- [x] Yes
- [ ] No

please see Figure 5

Explain why this information is not reported/not relevant.
